# *Mycobacterium tuberculosis* Adaptation in Response to Isoniazid Treatment in a Multi-Stress System That Mimics the Host Environment

**DOI:** 10.3390/antibiotics12050852

**Published:** 2023-05-05

**Authors:** Manita Yimcharoen, Sukanya Saikaew, Usanee Wattananandkul, Ponrut Phunpae, Sorasak Intorasoot, Chatchai Tayapiwatana, Bordin Butr-Indr

**Affiliations:** 1 Division of Clinical Microbiology, Department of Medical Technology, Faculty of Associated Medical Sciences, Chiang Mai University, Chiang Mai 50200, Thailand; manitayimcharoen@gmail.com (M.Y.); sukanya_saikaew@outlook.com (S.S.); usanee.anukool@cmu.ac.th (U.W.); ponrut.p@cmu.ac.th (P.P.); sorasak.in@cmu.ac.th (S.I.); 2 Division of Clinical Immunology, Department of Medical Technology, Faculty of Associated Medical Sciences, Chiang Mai University, Chiang Mai 50200, Thailand; chatchai.t@cmu.ac.th

**Keywords:** tuberculosis, isoniazid, stress response, adaptation, drug resistance

## Abstract

Isoniazid (INH) is an antibiotic that is widely used to treat tuberculosis (TB). Adaptation to environmental stress is a survival strategy for *Mycobacterium tuberculosis* and is associated with antibiotic resistance development. Here, mycobacterial adaptation following INH treatment was studied using a multi-stress system (MS), which mimics host-derived stress. Mtb H37Rv (drug-susceptible), mono-isoniazid resistant (INH-R), mono-rifampicin resistant (RIF-R), and multidrug-resistant (MDR) strains were cultivated in the MS with or without INH. The expression of stress-response genes (*hspX*, *tgs1*, *icl1*, and *sigE*) and lipoarabinomannan (LAM)-related genes (*pimB*, *mptA*, *mptC*, *dprE1*, *dprE2*, and *embC*), which play important roles in the host–pathogen interaction, were measured using real-time PCR. The different adaptations of the drug-resistant (DR) and drug-susceptible (DS) strains were presented in this work. *icl1* and *dprE1* were up-regulated in the DR strains in the MS, implying their roles as markers of virulence and potential drug targets. In the presence of INH, *hspX*, *tgs1*, and *sigE* were up-regulated in the INH-R and RIF-R strains, while *icl1* and LAM-related genes were up-regulated in the H37Rv strain. This study demonstrates the complexity of mycobacterial adaptation through stress response regulation and LAM expression in response to INH under the MS, which could potentially be applied for TB treatment and monitoring in the future.

## 1. Introduction

Tuberculosis (TB) is caused by *Mycobacterium tuberculosis* (Mtb). Worldwide, an estimated 10.6 million people were infected with TB and 1.6 million people died from TB in 2021 [1]. Treating Mtb infection is challenging due to the development of drug tolerance and drug resistance. During an infection as they are contained inside macrophages and within granulomas, mycobacteria encounter environmental stresses, such as nutrient limitation, hypoxia, acidic pH, nitric oxide, oxidative stress, and other host defense responses [2]. However, Mtb is a specialized pathogen that can adapt and persist within the host environment via stress response regulation [3,4]. The stress response of Mtb within the host environment requires the coordination of several events initiated by gene regulation and expression, resulting in physiological changes [5,6,7]. Environmental stress influences mycobacterial metabolic activity and induces a non-replicating state, allowing Mtb to persist for extended periods of time [8,9,10,11]. Moreover, mycobacterial adaptation and non-replicating state are associated with antibiotic tolerance, as presented in stress models and TB patients [8,9,10,12,13,14].

Isoniazid (INH) is a first-line drug regimen for TB treatment and a preventive therapy worldwide. INH inhibits the biosynthesis of mycolic acids and affects multiple targets, including nicotinamide adenine dinucleotide (NAD) metabolism, electron transport, nucleic acid synthesis, cell division, and efflux pumps [15,16]. The katG enzyme of mycobacteria activates INH, and the activated form binds to NADH-dependent enoyl-ACP reductase (InhA), which is required for mycolic acid synthesis, resulting in cell death [16]. INH effectively kills Mtb grown in vitro but is less active against mycobacteria grown in the host environment [17,18]. Resistance to INH increases the risk of additional drug resistance and leads to poor clinical outcomes [19]. Thus, studying the mechanism of mycobacterial adaptation in response to INH during an infection in a host is essential to improving our understanding of antibiotic resistance. INH treatment alters gene expression depending on the strains, growth phase, media, and culture conditions [18,20,21]. During the exponential phase, INH-treated Mtb regulates genes that encode enzymes in type II fatty acid synthesis, trehalose dimycolyl transferase, and other genes related to the isoniazid drug’s action [18,22]. In drug-resistant clinical isolates, efflux pump-related genes were induced in response to INH treatment [23]. In the fatty acid deficient model, an increase in the transcription of genes associated with glyoxylate shunt (*icl1*), methyl citrate cycle (*prpC* and *prpD*), and oxidative stress protection (*furA*, *katG*, *trxB1*, *ahpC*, and *ahpD*), together with slightly up-regulated dormancy survival regulator (*dosR*) gene, were observed in Mtb after exposure to INH [24]. Isocitrate lyase 1 (Icl1) encoded by *icl1* (Rv0467) is an enzyme in the glyoxylate shunt, and is associated with fatty acid metabolism, the critical pathway for Mtb during its infection in the host [25,26]. Moreover, Icl1 acts as an antioxidant, and Icl1-deficient strains are more susceptible to first-line drugs [24]. The DosR regulatory system is critical for mycobacterial adaptation and is required upon entry and during the dormancy stage [27]. Our previous work demonstrated that INH influences the expression of *hspX*, *tgs1*, and *sigE* with both up- and down-regulation, which varies among Mtb clinical isolates [21]. *hspX* (*acr,* Rv2031c) and *tgs1* (Rv3130c) are genes in the DosR regulatory system that encode heat shock protein HspX and triacylglycerol synthase, respectively [28,29]. HspX is required to maintain the viability of mycobacteria for long-term survival and to modulate growth during infections [13,30]. Under a stressful environment, Tgs1 causes triacylglycerol accumulation associated with the ability of mycobacteria to slow growth, reduce metabolic activity, and, consequently, increase antibiotic tolerance [12,31,32]. Extra cytoplasmic RNA polymerase sigma factor encoded by *sigE* (Rv1221) acts as a regulator in response to various stressors, including acid stress, oxidative stress, cell surface stress, high temperature, and during macrophage infection [33,34]. From the literature, the association between stress response and INH treatment suggests the role of INH as a stress inducer [21].

Mycobacterial cell wall remodeling is one of the strategies regulated in response to environmental stress [11,35]. Mycobacterial cell wall components are regulated depending on mycobacterial growth phases, culture medium, infection stages, and the presence of antibiotics [36,37,38]. Even though INH targets the mycolic acid of Mtb, it also affects other cell wall parts, such as lipoarabinomannan (LAM) [21]. The expression of genes involved in LAM synthesis (*pimB*, *mptA*, *mptC*, *dprE1*, *dprE2*, and *embC*) presents a unique pattern between drug-susceptible and drug-resistant strains following INH treatment [21]. LAM biosynthesis consists of three major parts: the mannosyl-phosphatidyl-myo-inositol (MPI) anchor, the mannan core, and the arabinomannan domain [39]. *pimB* (Rv2188c)-encoding mannosyltransferase is responsible for phosphatidylinositol mannoside (PIM) synthesis, the precursor for LAM production in the MPI anchor [40]. *mptA* (Rv2174) and *mptC* (Rv2181) encode mannosyltransferase and is responsible for the elongation and branching of the mannan backbone, respectively [41,42]. *dprE1* (Rv3790) and *dprE2* (Rv3791) encode enzymes that are required to produce decaprenylphosphoryl-D-arabinose (DPA), a precursor for arabinomannan domain formation [43]. *embC* (Rv3793) encode arabinosyltransferase for polymerizing arabinose into arabinan to form LAM [44]. LAM is an immunomodulator that causes phagosome maturation arrest, blocks oxidative response, modulates host cell signaling, influences cytokine production, alters T cell-mediated immunity, and affects antibody production [45,46]. Furthermore, LAM is modulated during infection, and a defect in LAM decreases disease progression [36,47]. However, the effect of INH treatment on LAM synthesis in Mtb under a hostile environment remains unclear.

**Figure 1:**
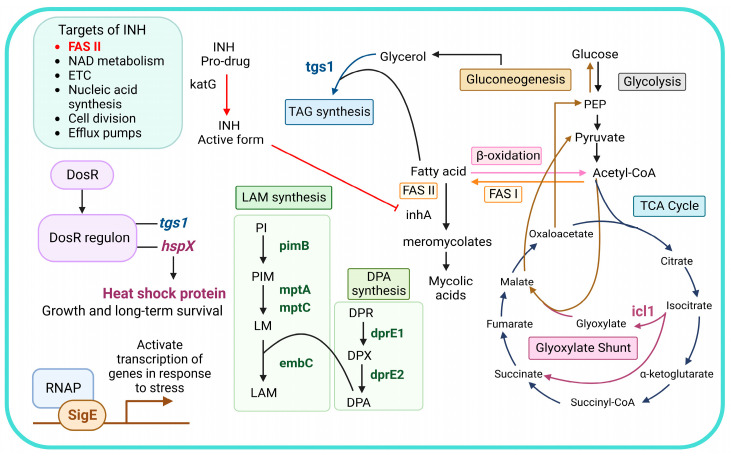
An overview of isoniazid’s mode of action and the roles of stress-response genes and LAM-related genes. Abbreviations: NAD, nicotinamide adenine dinucleotide; ETC, electron transport chain; DosR, dormancy survival regulator; DosR regulon, dormancy survival regulator regulon; RNAP, RNA polymerase; INH, isoniazid; TAG, triacylglycerol; PI, phosphatidylinositol; PIM, phosphatidylinositol mannoside; LM, lipomannan; LAM, lipoarabinomannan; DPR, decaprenylphosphoryl-D-ribose; DPA, decaprenylphosphoryl-D-arabinose; DPX, decaprenylphosphoryl-2-ketoribose; FASI, fatty acid synthase I; FAS II, fatty acid synthase II; PEP, phosphoenolpyruvate.

In this study, a multi-stress system (MS) was used as a model to study mycobacterial adaptation under stressful environments and in response to INH treatment, focusing on the expression of stress response and LAM-related genes. The MS mimicking the host environment, consisting of nutrient limitation, acidic pH, oxygen depletion, nitric oxide, and reactive nitrogen species, was modified from Gold B. et al. [48]. Mtb H37Rv (drug-susceptible strain) and clinical Mtb isolates with distinct genotypic and phenotypic drug resistance were cultivated in the MS and treated with or without INH at the C_Max_ concentration (6 µg/mL) for 30 min [20]. Based on the stress response initiated by gene regulation, the expression of stress-response genes (*hspX*, *tgs1*, *icl1*, and *sigE*) and LAM-related genes (*pimB*, *mptA*, *mptC*, *dprE1*, *dprE2*, and *embC*) were measured using real-time PCR. An overview of isoniazid’s mode of action and the roles of stress-response genes and LAM-related genes is presented in Figure 1.

## 2. Materials and Methods

### 2.1. Ethics and Biosafety Approval

This work was approved by the Institutional Ethics Committee and Biosafety Committee at Chiang Mai University (approval no.: AMSEC-63EM-028, CMUIBC A-0564001).

### 2.2. Isoniazid Stock Solution

Isoniazid (INH) (Sigma, St. Louis, MO, USA) at 1 mg/mL concentration was prepared in double distilled water and underwent filter sterilization using 0.2 µm nylon membrane filters. The INH stock solution was stored at −20 °C in dark vials until use.

### 2.3. Mycobacterial Strains

Mycobacterium tuberculosis H37Rv (ATCC 27294), a drug-susceptible strain, and three clinical Mtb isolates, including mono-isoniazid resistant (INH-R) Mtb, mono-rifampicin resistant (RIF-R) Mtb, and multidrug resistant (MDR) Mtb, were kindly provided by the Office of Disease Prevention and Control, 1 (Chiang Mai, Thailand). The drug-resistant genotypes of the clinical Mtb isolates were investigated [21]. Briefly, INH-R Mtb contains a single point mutation in the inhA promoter (C-15T), RIF-R Mtb harbors H526D RpoB mutation, and MDR Mtb has an amino acid change in KatG (S315T) and RpoB (D516V). Each Mtb strain was cultivated in a Lowenstein–Jensen medium (Biomedia, Nonthaburi, Thailand) at 37 °C in an aerobic culture condition for four weeks.

### 2.4. Culture Conditions

All mycobacteria were cultivated in nutrient-rich media (NR) for seven days to reach mycobacterial growth at the mid-log phase (OD_580_ = 0.4 to 0.7) prior to cultivation in the multi-stress system (MS) and multi-stress with INH treatment (MSI). Mtb was cultivated in the NR media containing Middlebrook 7H9 broth (M7H9) with 10% (*v*/*v*) OADC, 0.05% (*v*/*v*) tween 20, and 0.5% (*v*/*v*) glycerol, and incubated in an aerobic condition (20% O_2_ and 5% CO_2_) at 37 °C for seven days. The mycobacterial culture was centrifuged at 12,000× *g* at 20 °C for 20 min, and the supernatant was discarded. Then, each tube was washed with PBS-tyloxapol (2% *v*/*v* Tyloxapol in Dulbecco’s PBS without Ca^2+^ and Mg^2+^) [48] and centrifuged at 12,000× *g* at 20 °C for 20 min prior to being cultivated in the NR medium, the MS, and the MSI. For the NR medium, 30 mL of NR medium was added to the tube, adjusted to McFarland no. 1, and cultured at 37 °C in an aerobic culture (20% O_2_ and 5% CO_2_) for seven days. For the MS, the combination of nitric oxide, reactive nitrogen intermediates, acidic pH, oxygen depletion, and nutrient starvation that mimic a host-stressful environment was modified from a previous publication [48]. Mycobacteria were adjusted to McFarland no. 1 and cultivated in a multi-stress medium consisting of 0.5 g of KH_2_PO_4_, 0.5 g of MgSO_4_, 0.05 g of ammonium iron (III) citrate, 0.5% bovine serum albumin (BSA), 0.085% NaCl, 0.02% tyloxapol, 50 μM of butyrate, and 0.5 mM of NaNO_2_ at a pH of 5.0. Mycobacteria were incubated at 37 °C in an anaerobic jar for seven days. Oxygen was removed using the BD GasPak™ EZ anaerobe container system and monitored using methylene blue strips. For the MSI, 30 mL of the multi-stress media were added, adjusted to McFarland no. 1, and cultured in the multi-stress system for seven days. After complete incubation in the multi-stress system, INH at 6 µg/mL [20] (C_Max_ concentration) was added and incubated for 30 min.

### 2.5. Sample Preparation for Real-Time PCR Assay

Mycobacterial cells were harvested via centrifugation at 12,000× *g* at 4 °C for 20 min, and the supernatant was discarded. A NucleoSpin RNA extraction kit (MACHEREY-NAGEL, Düren, Germany) with an additional step was used for RNA extraction. Mycobacterial cells were lysed using a Tris-EDTA buffer (10mM of Tris-HCl and 1mM of EDTA; pH of 8.0) containing 2 mg/mL of lysozyme for 5 min. The mycobacterial cell suspension was transferred to a microcentrifuge tube containing 0.1 mm zirconia/silica beads (BioSpec, Bartlesville, OK, USA) and disrupted three times by an OMNI Bead Ruptor (OMNI, Kennesaw, GA, USA) at speed No. 2 for 1 min. RNA isolation was performed following the manufacturer’s protocols. The ReverTra Ace™ qPCR RT Master Mix with gDNA remover (Toyobo, Osaka, Japan) commercial kit was used for cDNA conversion. The RNA template was mixed with 4× DN Master Mix containing gDNA remover and incubated at 37 °C for 5 min. Then, 5× RT master mix was added and incubated at 37 °C for 15 min, at 50 °C for 5 min, and at 98 °C for 5 min. The optical density at 260 and 280 nm was measured using a microplate spectrophotometer (Biotek Epoch™, Santa Clara, CA, USA) to determine the purity and concentration. The RNA and cDNA samples were kept at −20 °C to −80 °C until use.

### 2.6. Real-Time PCR Targets Stress-Response Genes and LAM-Related Genes

The stress-response genes (*hspX*, *tgs1*, *icl1*, and *sigE*) and LAM-related genes (*pimB*, *mptA*, *mptC*, *dprE1*, *dprE2*, and *embC*) of Mtb in each culture condition were determined using real-time PCR assay and normalized using *sigA* (Rv2703c) gene [20,21]. Two hundred nanograms of the cDNA sample was used as a template, and the reaction was performed in the CFX96 Touch Real-Time PCR Detection System (Bio-Rad, Hercules, CA, USA). The 20 μL PCR reaction mixture consisted of 1xTHUNDERBIRDTM SYBR*^®^* qPCR Mix (Toyobo, Osaka, Japan) and 0.4 µM of the specific primers listed in Table 1. The specificity of all primers is demonstrated in the supplementary data. The PCR condition consisted of pre-denaturation at 95 °C for 5 min, followed by 37 cycles of 30 s at 95 °C, 30 s at 60 °C, and 30 s at 72 °C. The final extension was performed at 72 °C for 5 min. All samples were run in triplicate and performed in three independent experiments.

### 2.7. Calculation and Statistical Analysis

The relative gene expression was calculated using *sigA* (Rv2703c) for normalized gene expressions of target genes of Mtb that were cultivated in each culture condition [20,21]. The threshold cycle (CT) and 2^−ΔΔCT^ algorithm [49] were used to compare the expression of a target gene in two different conditions (MS vs. NR or MSI vs. MS) following the equations below [50].

For the expression of a target gene in MS compared with NR,

ΔCT_MS_ = CT (a target gene) *−* CT (*sigA*) (1)

ΔCT_NR_ = CT (a target gene) *−* CT (*sigA*) (2)

ΔΔCT = ΔCT_MS_ − ΔCT_NR_ (3)

2^−ΔΔCT^ = Normalized expression ratio (4)

The result obtained is the fold change of the target gene in MS relative to NR and is normalized to the expression of *sigA*.

For the expression of a target gene in MSI compared to MS,

ΔCT_MSI_ = CT (a target gene) *−* CT (*sigA*) (5)

ΔCT_MS_ = CT (a target gene) *−* CT (*sigA*) (6)

ΔΔCT = ΔCT_MSI_ − ΔCT_MS_ (7)

2^−ΔΔCT^ = Normalized expression ratio (8)

The result obtained is the fold change of the target gene in MSI relative to MS and is normalized to the expression of *sigA*.

The results represent the mean values of the relative expression levels ± SEM (standard error means). A one-way analysis of variance with Tukey’s multiple comparison was performed using GraphPad Prism v8. * indicates significant at ρ < 0.05; ** indicates significant at ρ < 0.01; *** indicates significant at ρ < 0.001; and **** indicates significant at ρ < 0.0001.

## 3. Results

### 3.1. Stress-Response Gene Expression of Mtb in a Multi-Stress System Is Differently Regulated among Strains

**Figure 2:**
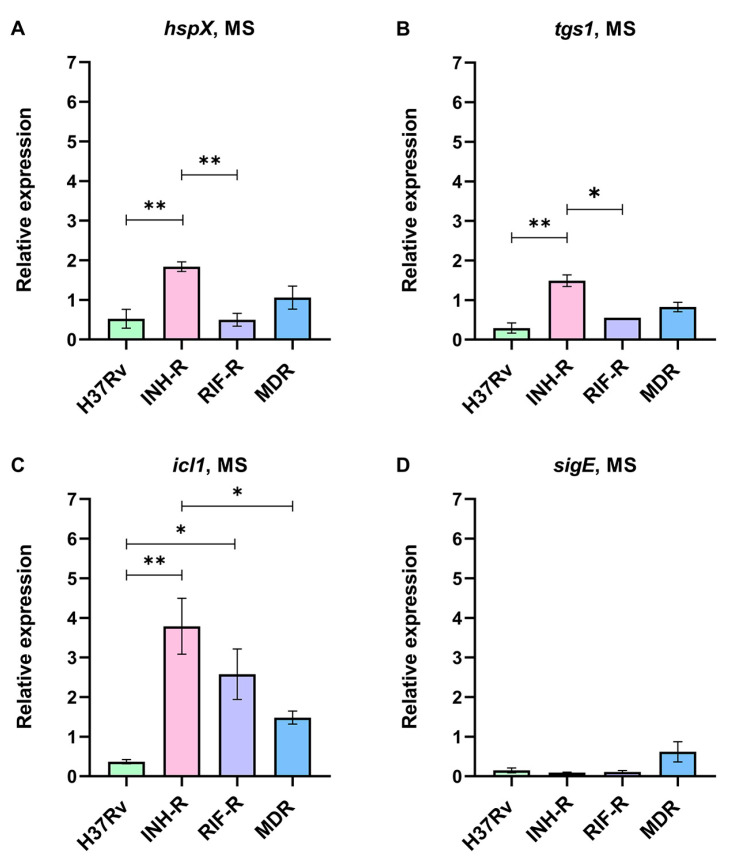
The relative expression of stress-response genes in four strains of Mtb in a multi-stress system (MS) compared to a nutrient-rich model (NR): (**A**) *hspX*, (**B**) *tgs1*, (**C**) *icl1*, and (**D**) *sigE*. * significant at ρ < 0.05; ** significant at ρ < 0.01 were determined by one-way analysis of variance with Tukey’s multiple comparison.

Mycobacterial cells were cultivated for seven days in a MS mimicking stress conditions in a host’s macrophages and granulomas. Due to the changes in the expressions of *hspX*, *tgs1*, *icl1*, and *sigE* in response to various stress, we measured these genes as stress-response markers in Mtb. The expression levels of stress-response genes normalized by *sigA* under the MS relative to the NR medium were calculated. We demonstrate that Mtb regulates stress-response genes, which are expressed at different levels among the four strains (Figure 2). Interestingly, the drug-susceptible strain (DS) could be differentiated from the drug-resistant strains (DR) using the expression patterns of stress-response genes. For the DR strains, *hspX* was up-regulated in the INH-R (1.84-fold) and MDR (1.06-fold) strains but down-regulated in the RIF-R strain (0.50-fold) (Figure 2A). The expression of *tgs1* decreased in the RIF-R (0.55-fold) and MDR (0.83-fold) strains but increased in the INH-R strain (1.49-fold) (Figure 2B). Moreover, *icl1* was remarkably up-regulated in the DR strains, including the INH-R (3.79-fold), RIF-R (2.58-fold), and MDR (1.48-fold) strains (Figure 2C), indicating an essential role of *icl1* for adaptation upon infection in the host. Several factors influence *sigE* expression, including bacterial growth phase, culture media, stressors, and incubation periods. In this work, after cultivating Mtb in the MS for seven days, *sigE* was down-regulated compared to the NR medium (Figure 2D). As we measured gene expression after cultivation in the MS for seven days (the early stress response), it might not reflect complete stress response. The distinct expression levels of stress-response genes of Mtb in the four strains under the MS support that mycobacterial stress response is model dependent [51]. The stress-response genes were not up-regulated in the DS strain but increased in the DR strains, suggesting that antibiotic resistance profile is one of the factors affecting mycobacterial adaptation in response to stress. As a result, the different mycobacterial adaptations could be one of the reasons why DR infection is more severe than DS infection.

### 3.2. A Single Treatment with INH in a Multi-Stress System Alters the Expression of Stress-Response Genes

**Figure 3:**
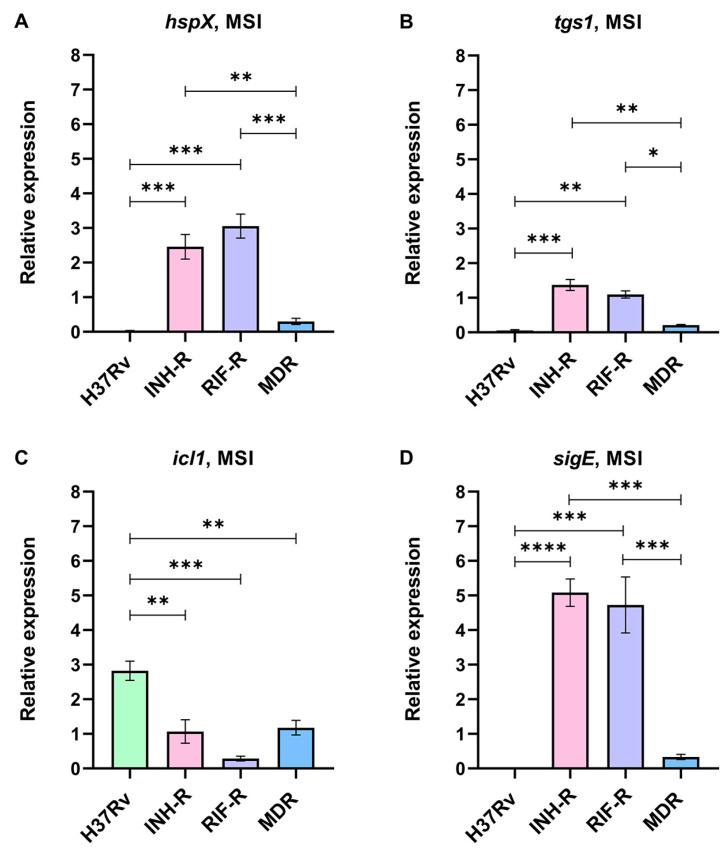
The relative expression of stress-response genes in four strains of Mtb in a multi-stress system with INH treatment (MSI) compared to a multi-stress system (MS) without INH treatment: (**A**) *hspX*, (**B**) *tgs1*, (**C**) *icl1*, and (**D**) *sigE*. * significant at ρ < 0.05; ** significant at ρ < 0.01; *** significant at ρ < 0.001; **** significant at ρ < 0.0001 were determined by one-way analysis of variance with Tukey’s multiple comparison.

The stress response of Mtb induced by INH under a MS mimicking host-derived stresses was investigated. Real-time PCR was performed using specific primers for *hspX*, *tgs1*, *icl1*, and *sigE* genes. The expression levels of stress-response genes normalized by *sigA* under the MSI relative to the MS were calculated. *hspX*, *tgs1*, and *sigE* were up-regulated in the mono-resistant strains, while *icl1* was strongly up-regulated in the DS strain in response to INH under the MS (Figure 3). For the INH-R strain, *hspX*, *tgs1*, and *sigE* were up-regulated at 2.46-fold, 1.37-fold, and 5.08-fold, respectively (Figure 3A,3B,3D). For the RIF-R strain, *hspX*, *tgs1*, and *sigE* were up-regulated at 3.06-fold, 1.10-fold, and 4.73-fold, respectively (Figure 3A,3B,3D). Furthermore, the statistical analysis showed that the INH-R and RIF-R strains significantly up-regulated *hspX*, *tgs1*, and *sigE* more than the H37Rv and MDR strains (Figure 3). *icl1* is an interesting drug target induced by INH under the MS, being significantly up-regulated in the DS strain (2.83-fold) and slightly up-regulated in the INH-R (1.07-fold) and MDR (1.18-fold) strains (Figure 3C). The stress response regulatory system is essential for survival and allows Mtb to shift into a quiescent state under diverse stress conditions [5,6]. Thus, the up-regulation of stress-response genes following INH treatment implies the role of INH as a stressor. In a quiescent state, mycobacteria withstand stresses and become antibiotic tolerant. Moreover, inappropriate antibiotic therapy is associated with drug resistance development during infection in the host [52]. Here, we demonstrated that stress-response genes were up-regulated in the mono-resistant strains and the DS strain in response to INH under the MS. These findings reflect the attempt of Mtb to adapt itself to survive in the presence of INH by regulating stress-response gene expression. This stress response may be critical for Mtb and an initial step toward additional antibiotic resistance. Furthermore, the results support that mycobacterial background (phenotypic and genotypic drug-resistance profiles) influences mycobacterial adaptation during INH treatment.

### 3.3. The Expression of LAM-Related Genes of Mtb with Distinct Drug-Resistance Profile in a Multi-Stress System

**Figure 4:**
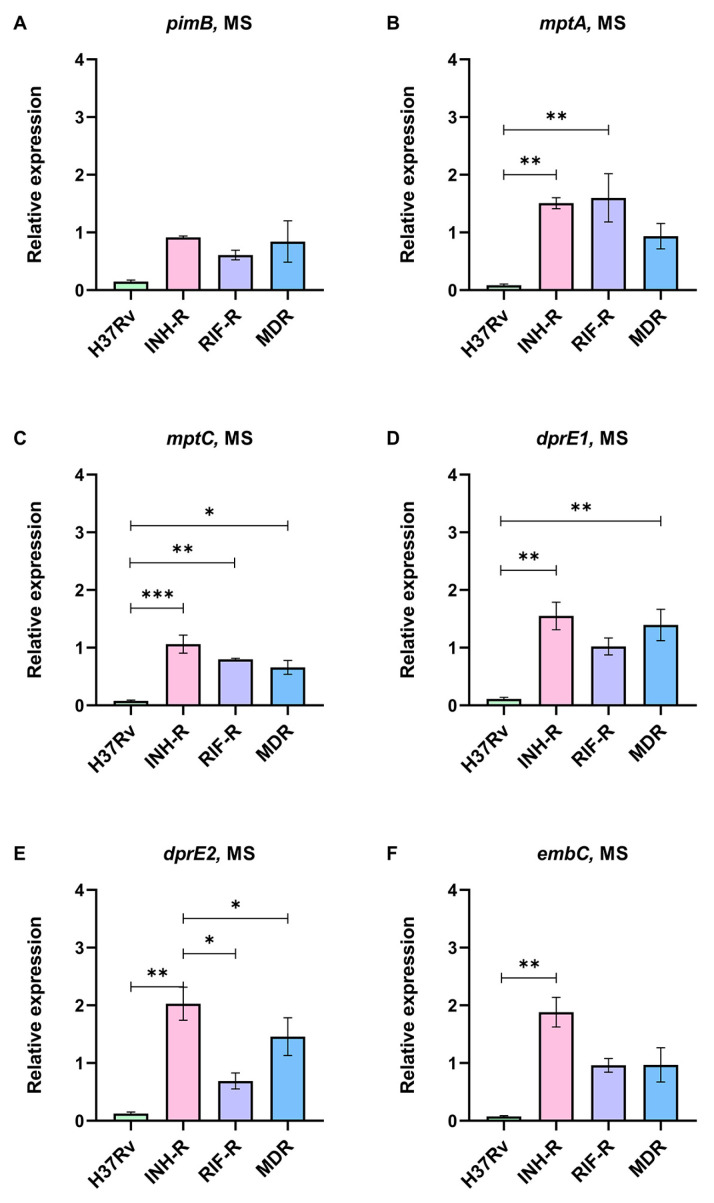
The relative expression of LAM-related genes in four strains of Mtb in a multi-stress system (MS) compared to a nutrient-rich model (NR): (**A**) *pimB*, (**B**) *mptA*, (**C**) *mptC*, (**D**) *dprE1*, (**E**) *dprE2*, and (**F**) *embC*. * significant at ρ < 0.05; ** significant at ρ < 0.01; *** significant at ρ < 0.001 were determined by one-way analysis of variance with Tukey’s multiple comparison.

Mtb cell wall plays a protective role in intracellular survival, and its components serve as virulence factors modulating the host’s immune response [53]. This work focuses on LAM regulation in a MS mimicking host stresses. The expression levels of genes responsible for LAM synthesis from the early to the last step were measured using real-time PCR with specific primers. The expression levels of LAM-related genes normalized by *sigA* under the MS relative to the NR medium were calculated. The early step of LAM synthesis requires *pimB* to form phosphatidylinositol mannoside (PIM), a precursor of LAM and a key molecule for host–pathogen interactions [40]. *pimB* was down-regulated in all Mtb cultivated under the MS conditions (Figure 4A), which correlated with a previous publication that reported a reduction in PIMs at the stationary phase of Mtb [36]. *mptA* and *mptC* are involved in the mannan backbone of lipomannan (LM) to form LAM and are related to the pathogenesis of Mtb [54]. We demonstrated that *mptA* was up-regulated in the INH-R (1.51-fold) and RIF-R (1.60-fold) strains, which was significantly greater than the DS strain (Figure 4B). Even though *mptC* was slightly increased only in the INH-R strain (1.06-fold), the expression of *mptC* in the DS strain was significantly lower than in the DR strains (Figure 4C). The different expressions of *mptA* and *mptC* between the DR and DS strains in response to stresses support the role of these genes in the pathogenicity of Mtb during infection. *dprE1* and *dprE2* are required to epimerize decaprenylphosphoryl ribose (DPR) to DPA, a precursor for the arabinomannan domain [43]. *dprE1* and *dprE2* were up-regulated in the INH-R and MDR strains (Figure 4D,E). Moreover, the expression of *dprE1* in INH and MDR strains was significantly up-regulated compared to the DS strain (Figure 4D). In the RIF-R strain, *dprE1* was slightly up-regulated (1.02-fold), but *dprE2* expression decreased (0.69-fold) (Figure 4D,E). The similar expression patterns of *dprE1* and *dprE2* in the INH-R and MDR strains suggest the involvement of INH resistance and mycobacterial adaptation to stress via DPA production. embC, which is required to form the arabinan structure of LAM [44], tends to be stable in the RIF-R and MDR strains (Figure 4F). However, *embC* was clearly up-regulated in the INH-R strain (1.88-fold), and its expression level was significantly greater than in the DS strain (Figure 4F). We highlight that LAM-related genes are mainly up-regulated in the INH-R strain, *dprE1* is up-regulated in the DR strains, and *mptA* is up-regulated in the mono-resistant strains. These distinct patterns indicate the regulation of LAM under stressful environments, which varies among the strains.

### 3.4. Mycobacterial Adaptation via LAM-Related Gene Expression Induced by INH Treatment under a Multi-Stress System

**Figure 5:**
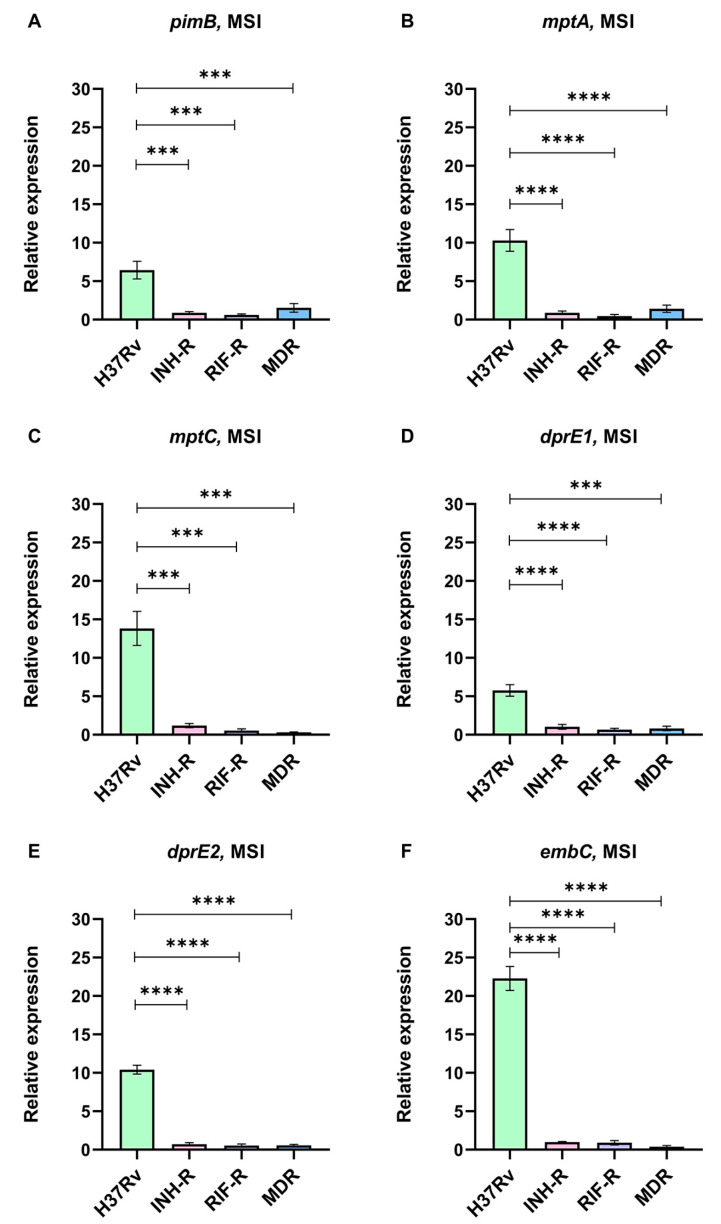
The relative expression of LAM-related genes in four strains of Mtb in a multi-stress system with INH treatment (MSI) compared to a multi-stress system (MS) without INH treatment: (**A**) *pimB*, (**B**) *mptA*, (**C**) *mptC*, (**D**) *dprE1*, (**E**) *dprE2*, and (**F**) *embC*. *** significant at ρ < 0.001; **** significant at ρ < 0.0001 were determined by one-way analysis of variance with Tukey’s multiple comparison.

We cultivated Mtb in the MS and treated it with INH to investigate the initial mycobacterial adaptation, which occurs via LAM regulation. The expressions of LAM-related genes normalized by *sigA* under the MSI relative to the MS were calculated. Although LAM-related genes were not up-regulated in the DS strain under the MS, LAM-related genes were dramatically up-regulated following INH treatment (Figure 5). Interestingly, the expressions of LAM-related genes of the DS strain were significantly greater than those of the INH-R, RIF-R, and MDR strains (Figure 5). For the DR strain, the expressions of LAM-related genes tend to be stable or change slightly in response to the INH treatment compared to the MS without INH. The changes in LAM-related gene expression suggest that LAM regulation may be necessary for mycobacterial adaptation during INH treatment, particularly for the DS strain.

## 4. Discussion

**Figure 6:**
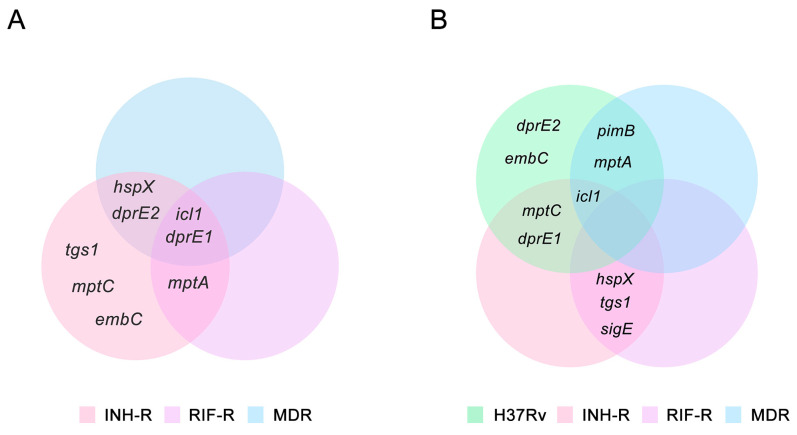
The Venn diagrams demonstrate stress-response genes and LAM-related genes of the H37Rv, INH-R, RIF-R, and MDR strains, which are up-regulated under the MS and MSI conditions: (**A**) a multi-stress system (MS) compared to a nutrient-rich model (NR) and (**B**) a multi-stress system with INH treatment (MSI) compared to a multi-stress system (MS) without INH treatment.

**Figure 7:**
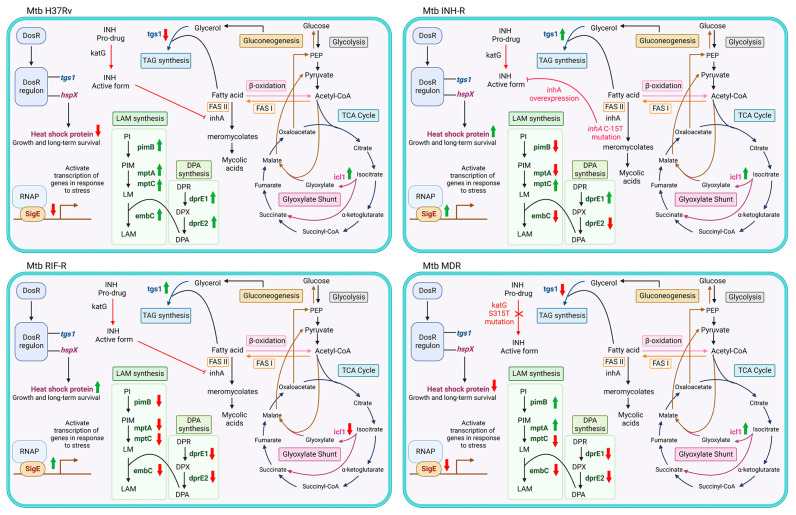
A summary of the expression of LAM-related genes and stress-response genes in response to INH under the MS. The red arrows represent down-regulation and the green arrows represent up-regulation when compared between the MSI and MS. Abbreviations: DosR, dormancy survival regulator; DosR regulon, dormancy survival regulator regulon; RNAP, RNA polymerase; INH, isoniazid; TAG, triacylglycerol; PI, phosphatidylinositol; PIM, phosphatidylinositol mannoside; LM, lipomannan; LAM, lipoarabinomannan; DPR, decaprenylphosphoryl-D-ribose; DPA, decaprenylphosphoryl-D-arabinose; DPX, decaprenylphosphoryl-2-ketoribose; FASI, fatty acid synthase I; FAS II, fatty acid synthase II; PEP, phosphoenolpyruvate.

Upon infection, Mtb experiences diverse stress conditions that drive transcriptional changes responsible for adaptation and promote survival [3]. The stressful environment reduces mycobacterial metabolic activity and induces a non-replicating state, where Mtb acquires antibiotic tolerance [12,51]. Thus, it is necessary to explore mycobacterial adaptation during infection and treatment. In this work, a MS comprising host stress was used to investigate the stress response of Mtb following INH treatment. The MS without INH treatment represents the host environment before starting treatment, which was used to investigate the effect of stress on Mtb. The MSI represents TB patients initially treated with INH, which was used to elucidate the stress response induced by INH. We measured the expressions of genes that are related to stress response and provided the link between mycobacterial adaptation through LAM regulation under host stress. The adaptability of Mtb in response to host stress varies among the strains and causes different infection outcomes [4,55], Mtb with distinct drug-resistance profiles were studied. The gene expression patterns in the MS compared to the NR medium and in the MSI compared to the MS are presented in Figure 6A and 6B, respectively. We demonstrated the different expression patterns of stress-response genes under the MS conditions among the DR and DS strains (Figure 6A). Importantly, the INH treatment appeared to affect the expression of stress-response genes, implying the role of INH as a stressor that promotes the stress response of mycobacteria (Figure 6B). A summary of the expression of LAM-related genes and stress-response genes in response to INH under the MS is presented in Figure 7. *hspX* and *tgs1* are genes in the DosR regulatory system, which play a role in mycobacterial persistence and are essential for modulating cellular metabolism upon entry into and during dormancy [13,56]. *hspX* and *tgs1* are regulated in hypoxia, multiple-stress model, macrophage infection, and dormancy [12,57,58,59,60]. However, the role of the DosR regulatory system in virulence is model dependent [61]. In this study, *hspX* and *tgs1* were up-regulated in the INH-R strain, down-regulated in the RIF-R strain, and slightly changed in the MDR strain under the MS conditions. Apart from the DosR regulatory system, *sigE* is modulated in response to acidic pH, oxidative stress, and cell wall stress, which are required for intracellular survival inside macrophages [62,63]. Additionally, SigE regulates the expression of several genes involved in lipid metabolism and cell wall integrity [63,64]. Interestingly, we found that *hspX*, *tgs1*, and *sigE* were up-regulated in the INH-R and RIF-R strains following INH treatment under the MS. Taken together, the up-regulation of these genes in response to INH under the MS may be necessary to maintain survival, regulate cellular processes, and provide Mtb entry into a quiescent state. This may lead to treatment failure associated with additional drug-resistance development in TB patients infected with mono-drug resistant strains. However, these genes were not upregulated following INH treatment in the H37Rv and MDR strains, indicating the involvement of other mechanisms. The regulation of fatty acid metabolism contributes to the virulence and pathogenicity of Mtb during infection within the host [26]. Icl1 is an enzyme involved in fatty acid metabolism [25], promoting persistence in the host and mediating broad antibiotic tolerance of Mtb [6,24,65]. Increased expression of *icl1* has been demonstrated in an acidic environment [66,67,68], in the Wayne dormancy model (hypoxia) [57], in macrophage infection [13], and in a multiple-stress model [12]. Moreover, *icl1* expression levels vary based on mycobacterial strains, carbon sources, and culture periods [6,26,69]. Likewise, we demonstrated that *icl1* was up-regulated in the drug-resistant strains under the MS conditions (Figure 6A) and up-regulated in the H37Rv, INH-R and MDR strains after INH treatment (Figure 6B). The critical role of *icl1* for adaptation and its expression levels in this work indicate that it is an attractive virulence marker and a potential target for TB treatment [70].

We measured the expression of LAM-related genes of Mtb cultivated in a MS that mimicked the host environment with or without INH. Previous publication has demonstrated that LAM regulation is involved in mycobacterial adaptation in a nutrient starvation model, and genes involved in LAM synthesis are up-regulated at the stationary phase [71]. Similarly, LAM-related genes were regulated in the MS, as presented in the different levels between the four strains (Figure 4). *dprE1* is responsible for LAM synthesis and maintaining the permeability of mycobacterial cell wall [72]. We found that *dprE1* gene was up-regulated in all DR strains under the MS, suggesting its protective role during infection and showing that it might be involved in the virulence of drug-resistant strains. The relative expression of LAM-related genes in the presence of INH was compared in each strain, but the result from the DR strain of Mtb showed that it was not significantly different. Surprisingly, LAM-related genes in the H37Rv strain were dramatically up-regulated following the INH treatment under the MS (Figure 5). Among the LAM-related genes, *embC* was the most up-regulated gene in the H37Rv strain in response to the INH treatment. *embC* is an essential gene for LAM synthesis and serves as a target for ethambutol (EMB) [73]. It has been suggested that the size of LAM depends on the activity of EmbC and an overexpression of *embC* results in EMB resistance [74]. Furthermore, Bacon et al. reported that during starvation, Mtb promotes the expression of *embC* and, consequently, increases the arabinose content of LAM [71]. Thus, the up-regulation of LAM-related genes may be a defense mechanism of the DS strain to maintain viability during isoniazid treatment. Moreover, the distinct gene expression patterns among the four Mtb strains suggest that drug-resistance profile determines the factors influencing cell wall remodeling after antibiotic exposure. It is inevitable to have some limitations in this study. Firstly, the MS condition contains the main stress that Mtb would encounter in a host but does not include all host-derived stresses. Secondly, we used Mtb clinical samples that are major DR strains. Finally, we focused on some stress-response genes and LAM-related genes, but there are many genes that are involved in response to a stressful environment.

## 5. Conclusions

This study investigated the stress response of Mtb strains with distinct drug-susceptibility profiles (H37Rv, INH-R, RIF-R, and MDR) following INH treatment under a MS that mimics a host’s environment. The expression levels and patterns of stress-response genes (*hspX*, *tgs1*, *icl1*, and *sigE*) and LAM-related genes (*pimB*, *mptA*, *mptC*, *dprE1*, *dprE2,* and *embC*) were distinct between the drug-susceptible and drug-resistant strains. In the absence of INH, *icl1* and *dprE1* were predominantly up-regulated in the INH-R, RIF-R, and MDR strains under the MS conditions, suggesting their essential roles during infection and as possible drug targets for TB treatment. However, in the presence of INH, *hspX*, *tgs1,* and *sigE* were significantly up-regulated in the INH-R and RIF-R strains, while *icl1* and LAM-related genes were significantly up-regulated in the H37Rv strain. These findings highlight the role of INH as a stressor and emphasize the importance of proper and consistent use of antibiotics. Moreover, the regulation of LAM-related genes is involved in stress response during treatment with INH and may be crucial for mycobacterial adaptation and persistence in the host. These results provide insights into the mechanisms underlying drug resistance and suggest potential targets for the development of new therapies.

## Figures and Tables

**Table 1:** The list of specific primers for the expression analysis of stress-response genes and LAM-related genes.

Target	Forward (F) and Reverse (R) Primers 5′ → 3′	Amplicon Length (bp)	Reference
*hspX* (Rv2031c)	F: CGACAAGGACGTCGACATTA R: CCTTGTCGTAGGTGGCCTTA	173 bp	[21]
*tgs1* (Rv3130c)	F: TAGCTGGGCTCTCCGATGAA R: ATTGACACGGAATCCACCCC	107 bp	[21]
*icl1* (Rv0467)	F: GTTGGCCTCTGAGAAGAAGTG R: CAGCGTGATGAACTGGAACT	493 bp	This work *
*sigE* (Rv1221)	F: GCAGTGCAAATTCGGAGGAT R: ATTGGTCAGACGGCTCCA	114 bp	[21]
*pimB* (Rv2188c)	F: GCGGTAGGTATTCCAACGAAG R: TATGCACCGCAGTGGAAAG	394 bp	[21]
*mptA* (Rv2174)	F: CGGTTGATTTGGCTACAGCG R: CGTAAGGATCCAGACCGTCG	435 bp	[21]
*mptC* (Rv2181)	F: ATAGCCCTCAAACTCACCCC R: TCATCGCCAATCGTCAACC	245 bp	[21]
*dprE1* (Rv3790)	F: TATCCACTCCATTGACGCCG R: ATGATGATGCCGGTGAGACC	318 bp	[21]
*dprE2* (Rv3791)	F: GACAGCCACCCGAAGATGAT R: AACCCCAGGTAAAACCCGTC	302 bp	This work *
*embC* (Rv3793)	F: CCGACAAAGTGGACCCATCA R: ACCGAAGTTGGACACGTACC	195 bp	This work *
*sigA* (Rv2703)	F: GTCGGAGGCCCTGCGTCAAG R: AGGCCAGCCTCGATCCGCTT	147 bp	[20]

* primers were designed in this study using the NCBI primer blast.

## Data Availability

Not applicable.

## Supplementary Materials

The following supporting information can be downloaded at: https://www.mdpi.com/article/10.3390/antibiotics12050852/s1.
