# Supplementary material for: Mycobacterium tuberculosis Adaptation in Response to Isoniazid Treatment in a Multi-Stress System That Mimics the Host Environment
Source: Antibiotics (Basel). 2023 May 5;12(5):852. doi: 10.3390/antibiotics12050852 (PMC10215434; doi:10.3390/antibiotics12050852)
Supplement: Supplementary file 1 [file antibiotics-12-00852-s001.zip › antibiotics-2357981-supplementary.pdf]

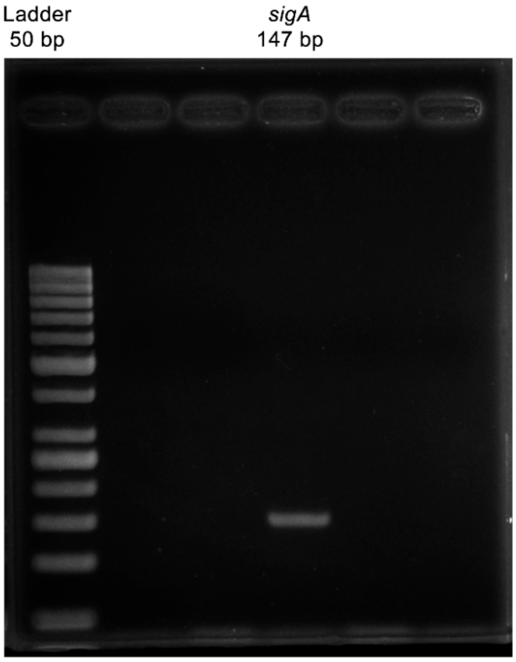

Supplementary Figure S1. PCR product using primers target *sigA* gene.

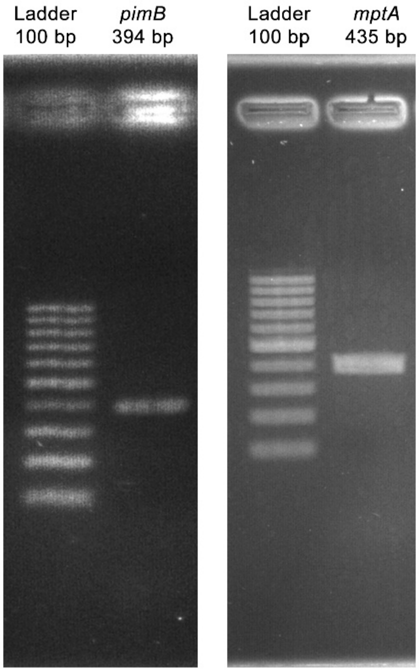

Supplementary Figure S2. PCR products using primers target *pimB* and *mptA* genes.

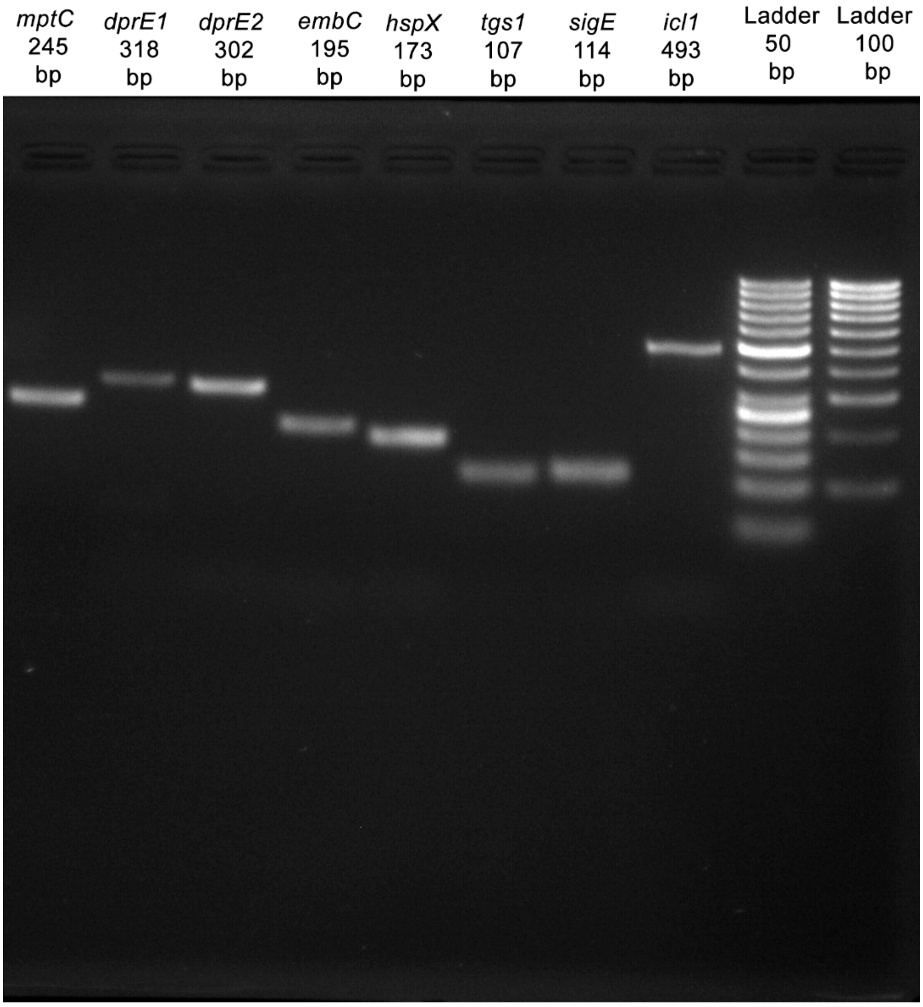

Supplementary Figure S3. PCR products using primers target *mptC, dprE1, dprE2, embC, hspX, tgs1, sigE,* and *icl1* genes.

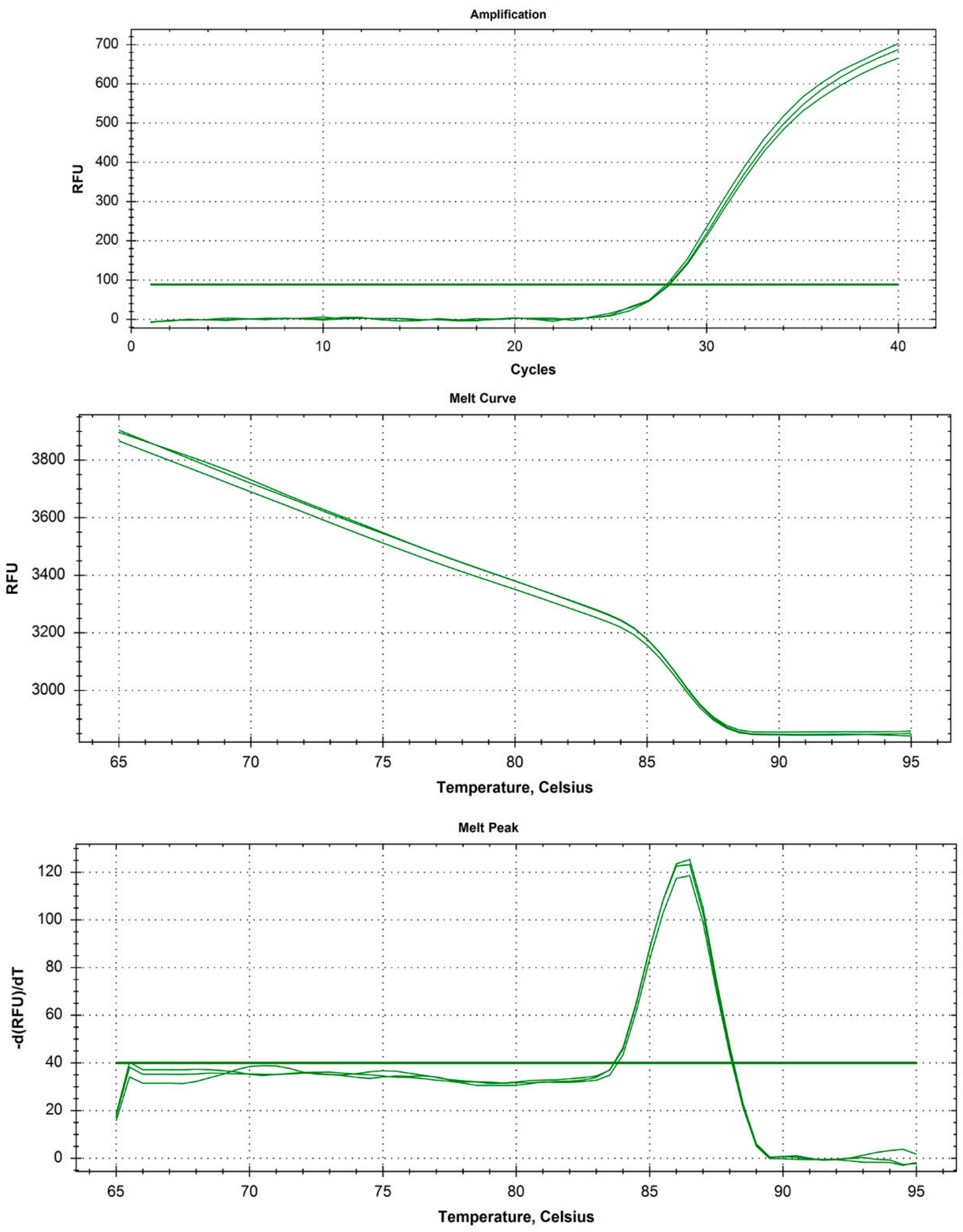

Supplementary Figure S4. PCR amplification curve and melt curve analysis of *hspX* gene of Mtb H37Rv.

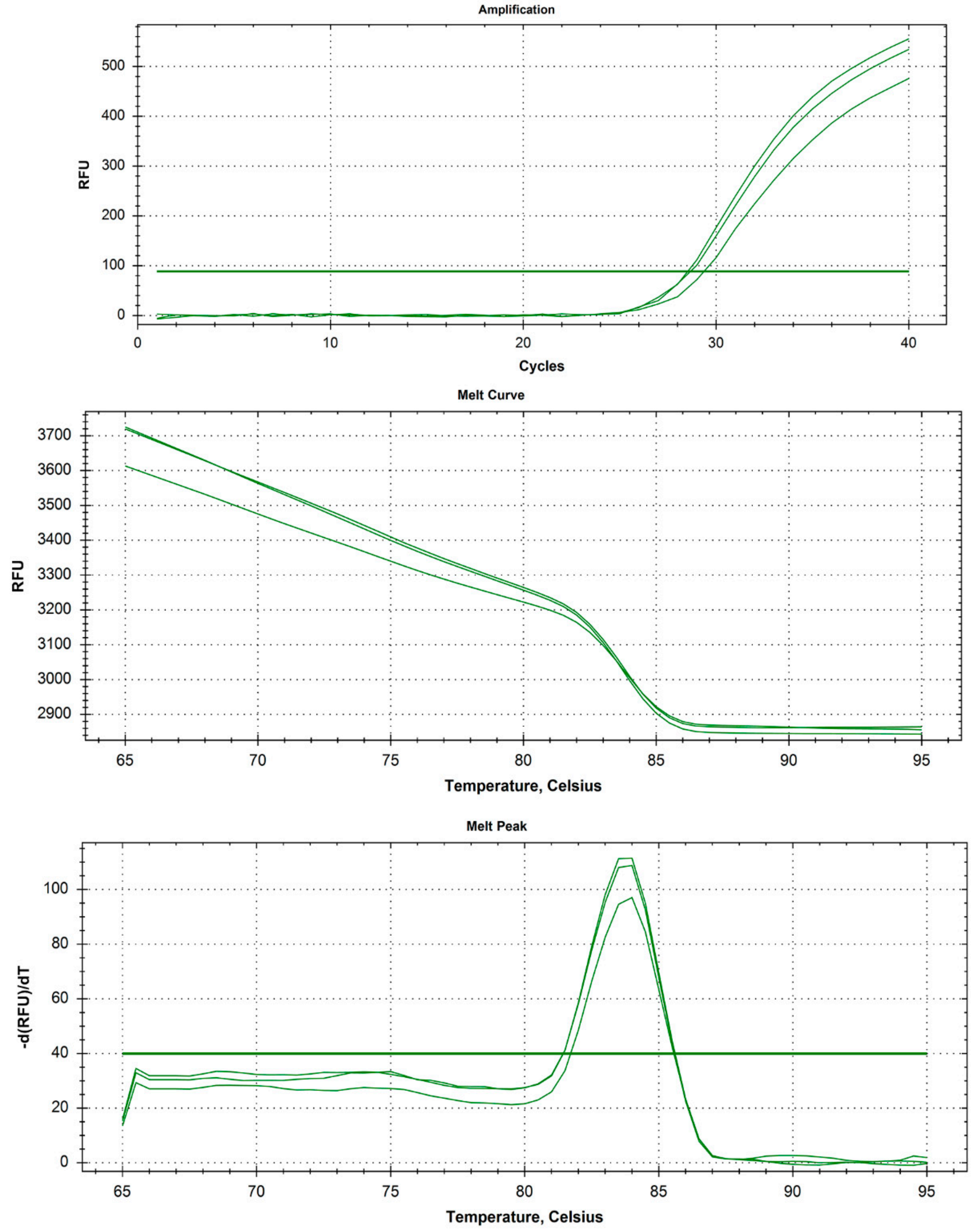

Supplementary Figure S5. PCR amplification curve and melt curve analysis of *tgs1* gene of Mtb H37Rv.

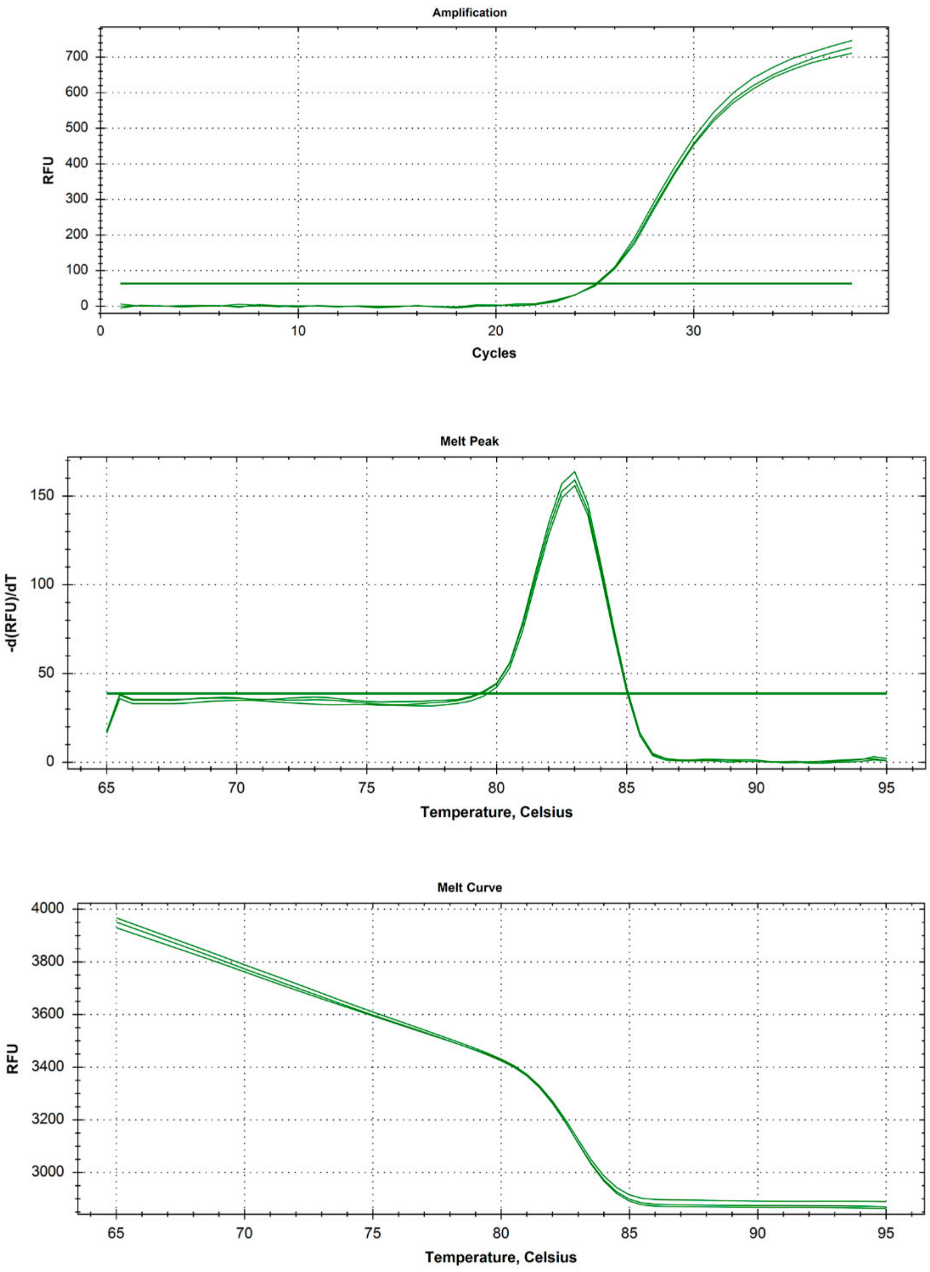

Supplementary Figure S6. PCR amplification curve and melt curve analysis of *sigE* gene of Mtb H37Rv.

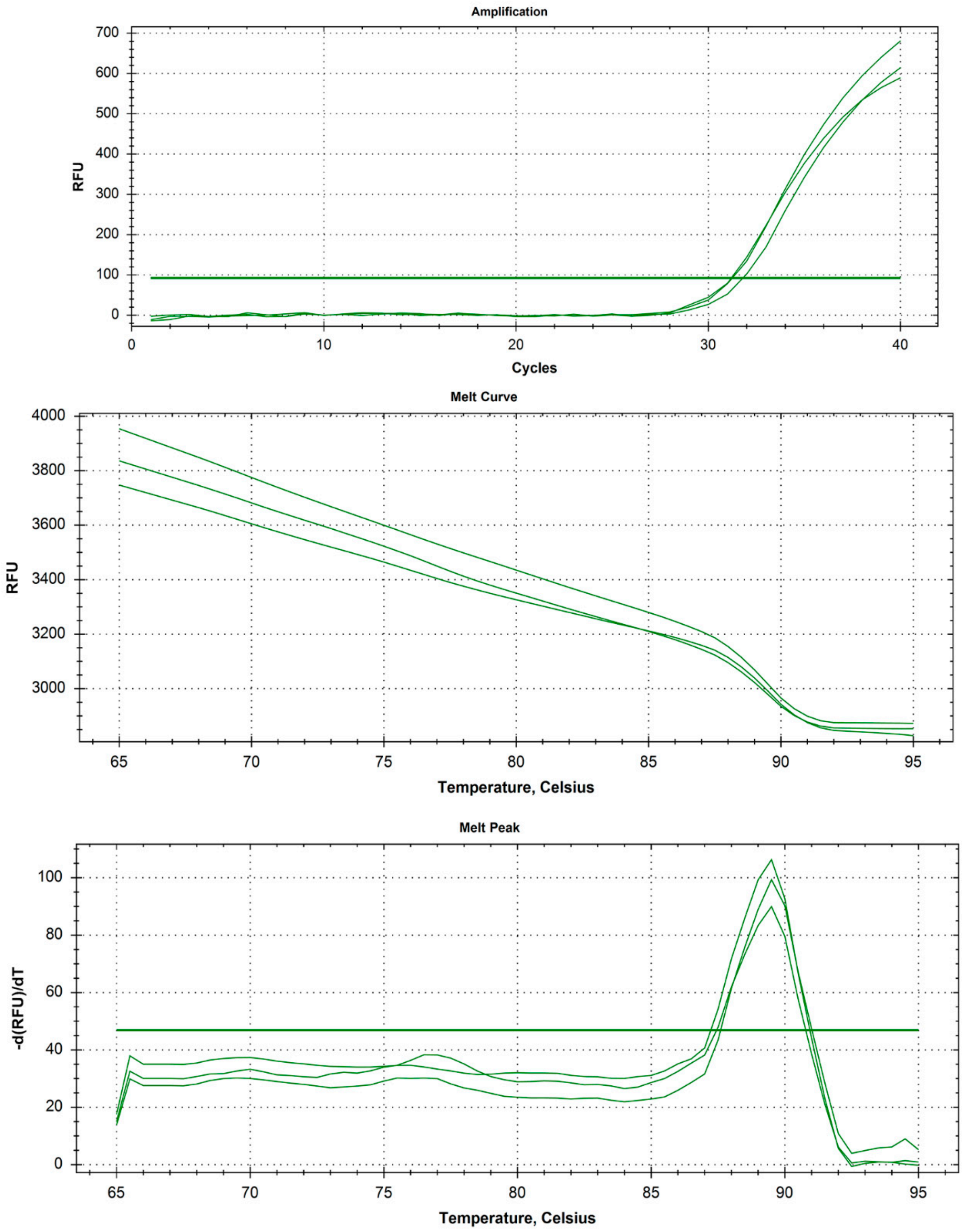

Supplementary Figure S7. PCR amplification curve and melt curve analysis of *icl1* gene of Mtb H37Rv.

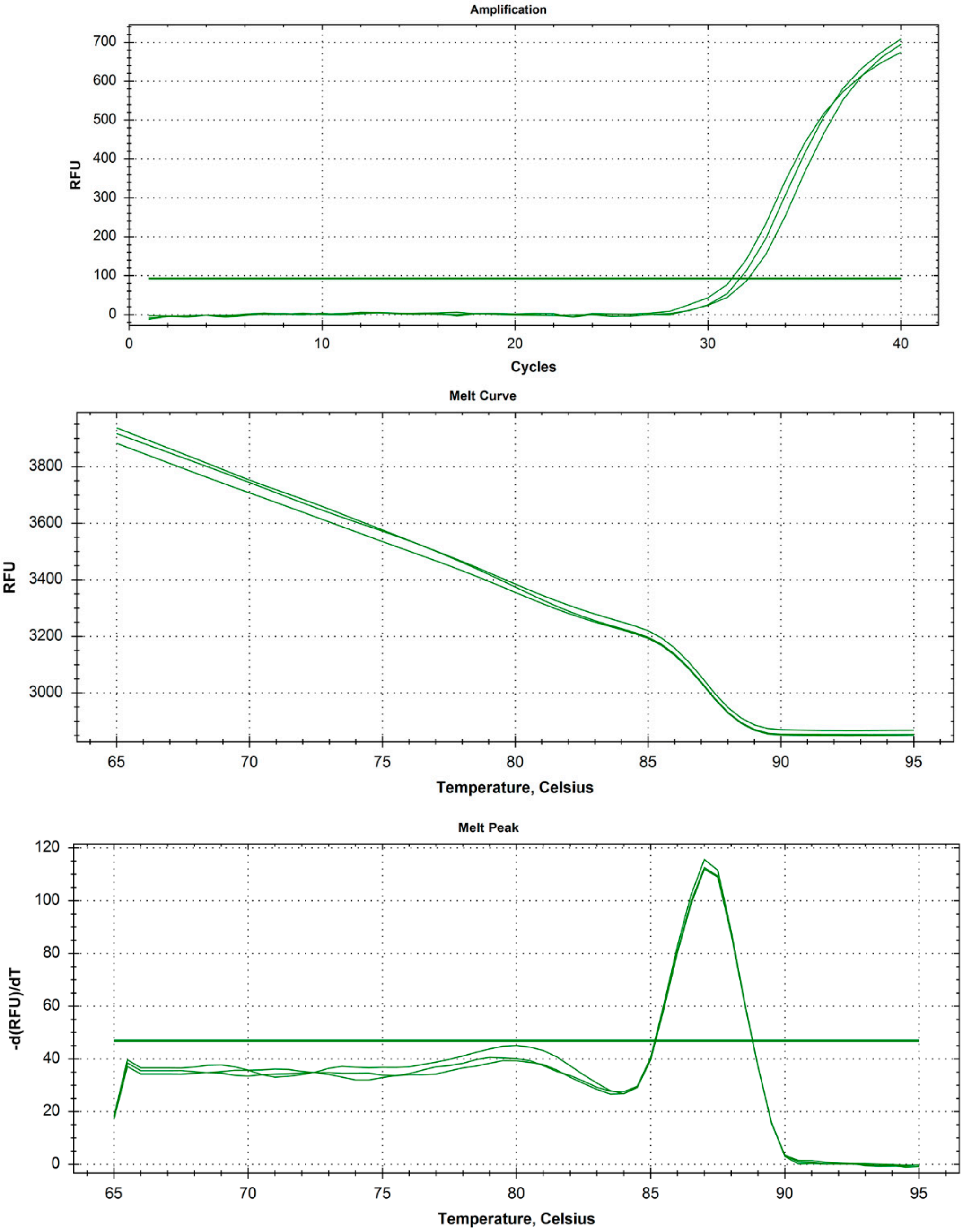

Supplementary Figure S8. PCR amplification curve and melt curve analysis of *sigA* gene of Mtb H37Rv.

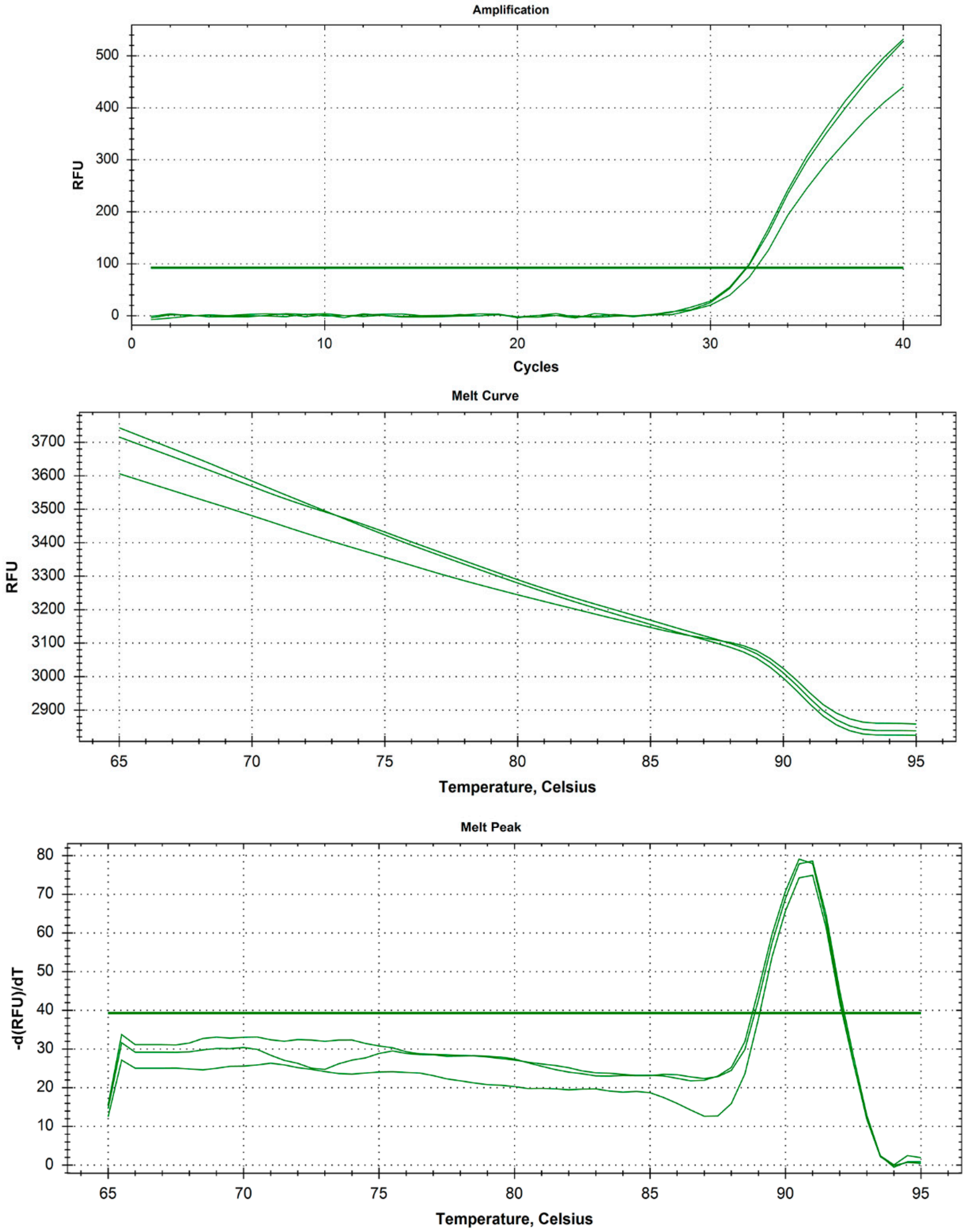

Supplementary Figure S9. PCR amplification curve and melt curve analysis of *pimB* gene of Mtb H37Rv.

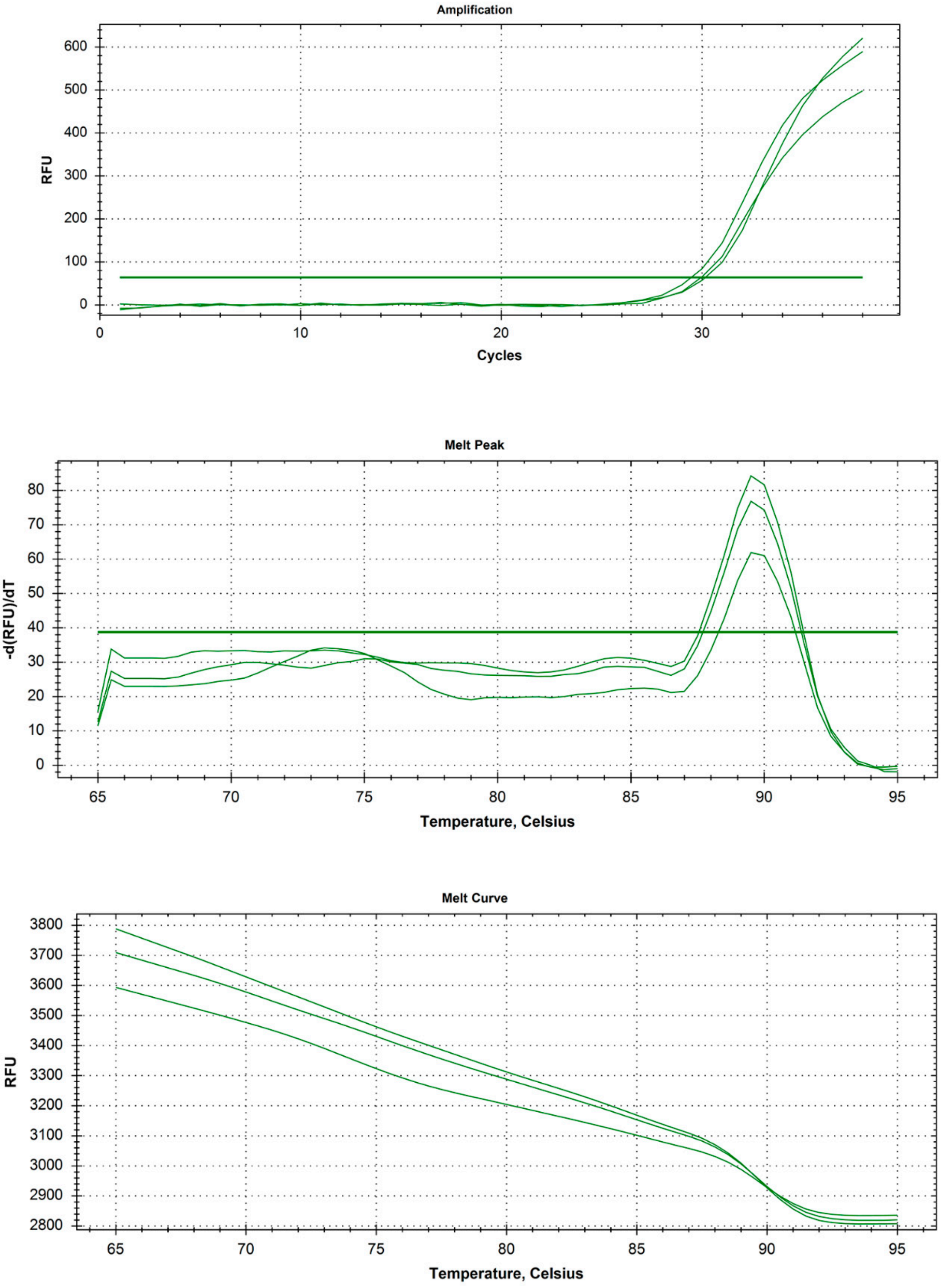

Supplementary Figure S10. PCR amplification curve and melt curve analysis of *mptA* gene of Mtb H37Rv.

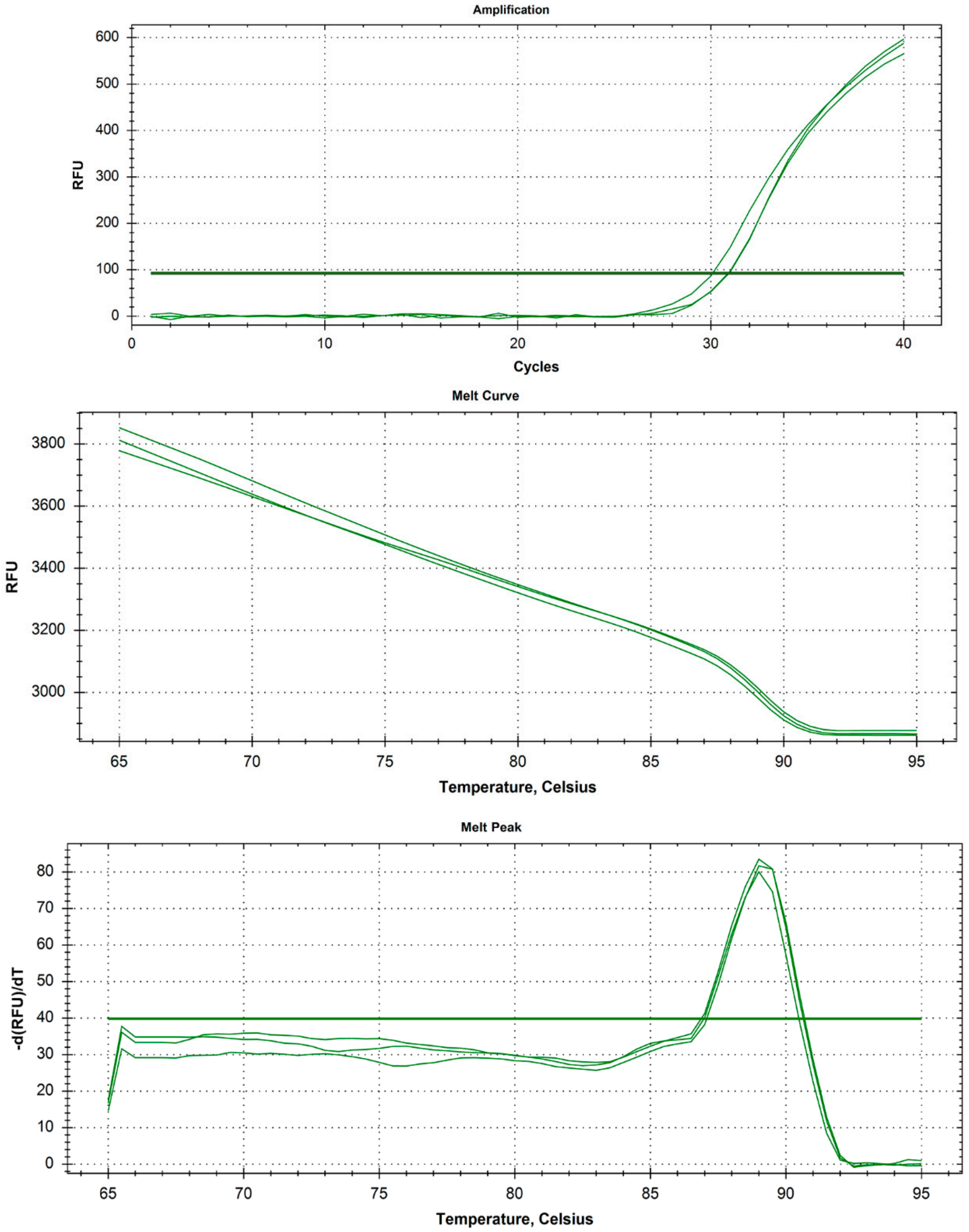

Supplementary Figure S11. PCR amplification curve and melt curve analysis of *mptC* gene of Mtb H37Rv.

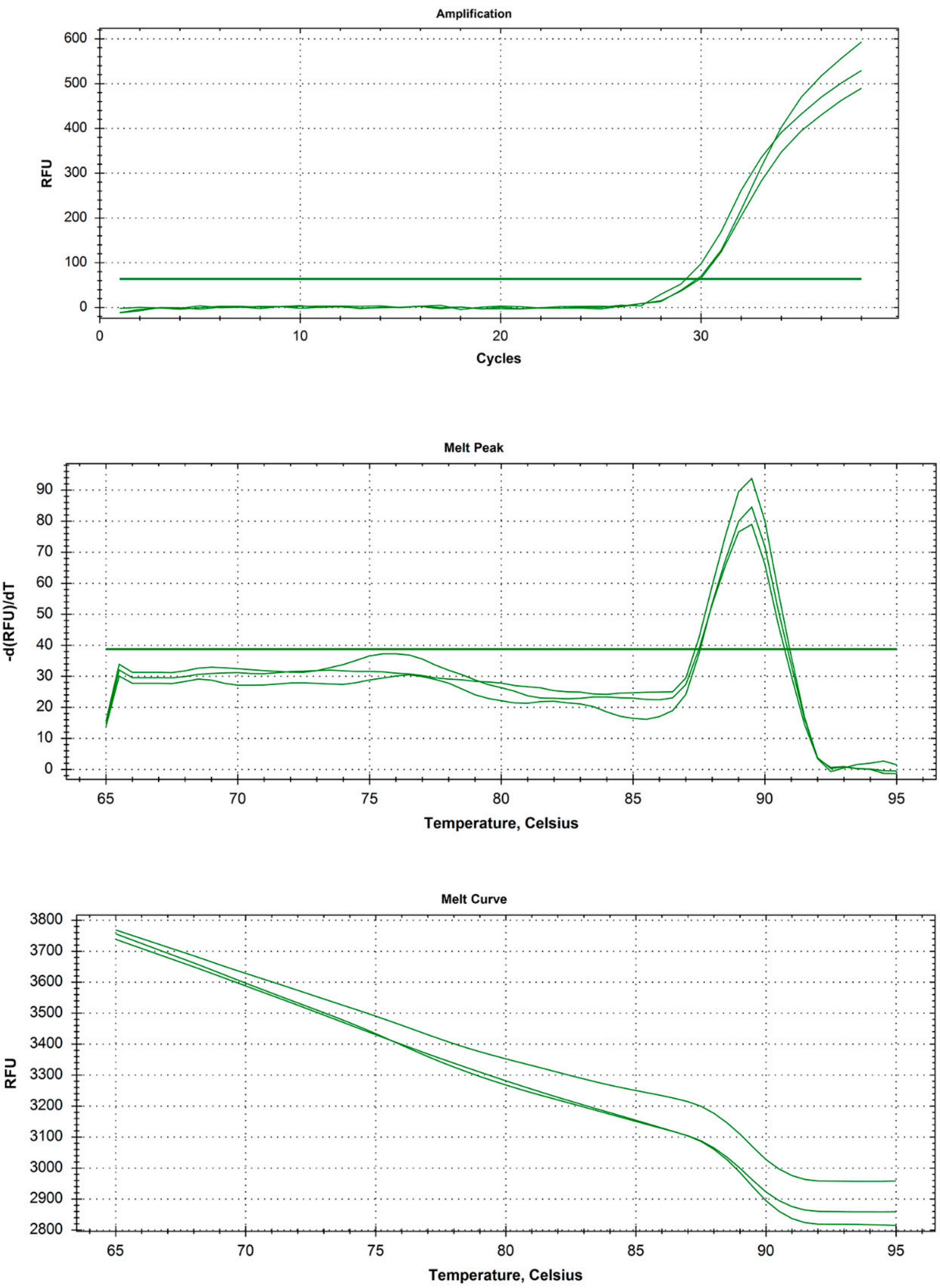

Supplementary Figure S12. PCR amplification curve and melt curve analysis of *dprE1* gene of Mtb H37Rv.

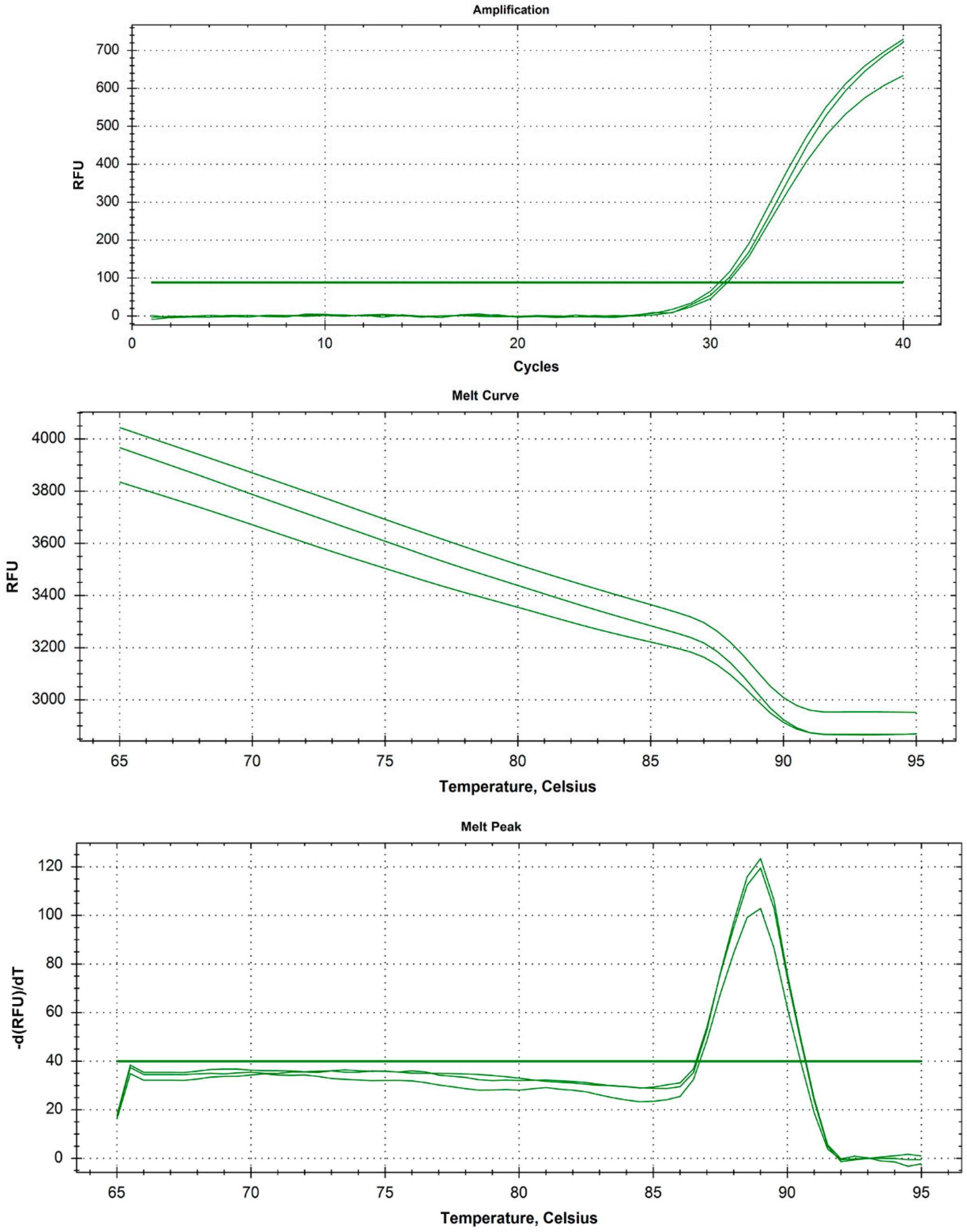

Supplementary Figure S13. PCR amplification curve and melt curve analysis of *dprE2* gene of Mtb H37Rv.

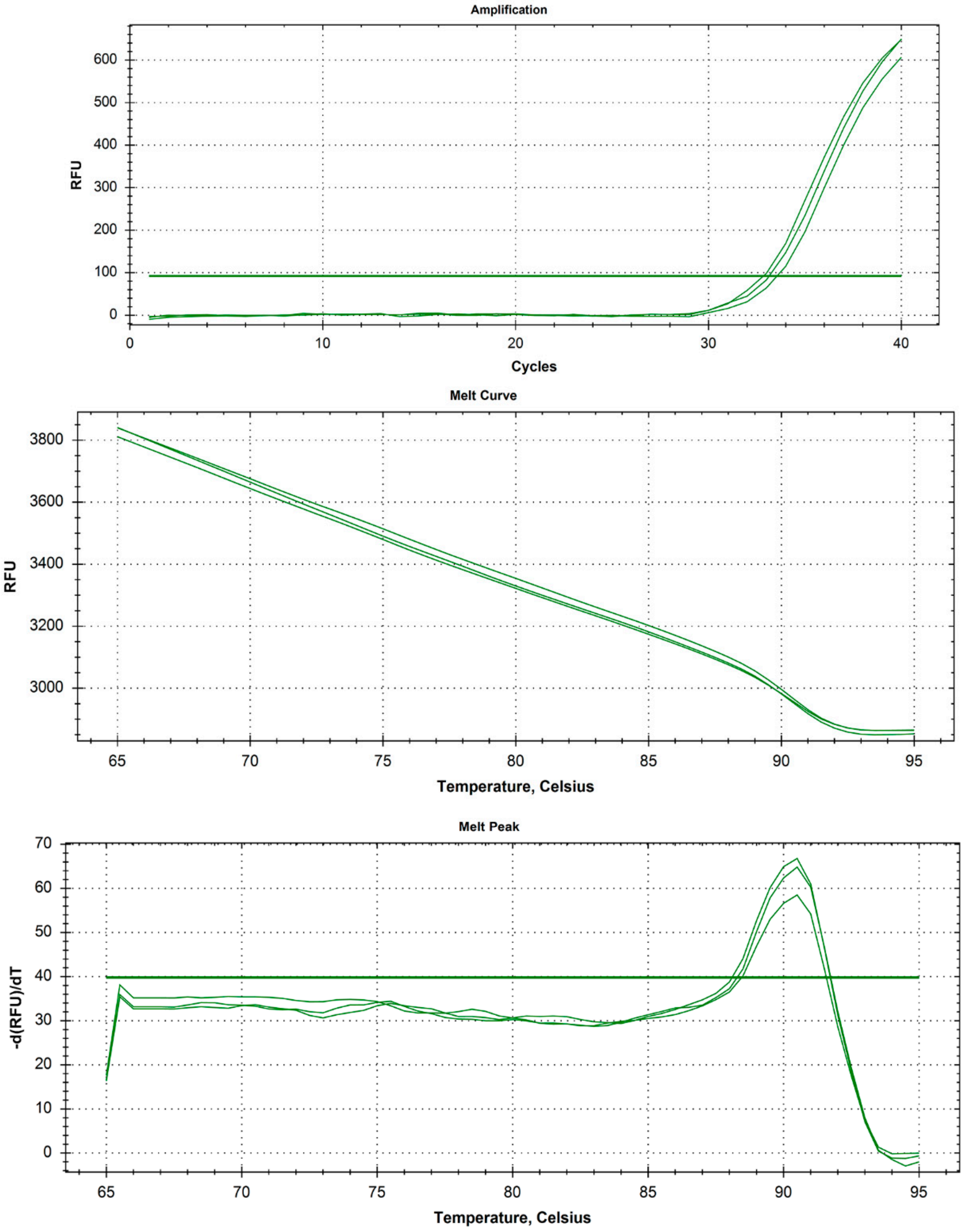

Supplementary Figure S14. PCR amplification curve and melt curve analysis of *embC* gene of Mtb H37Rv.

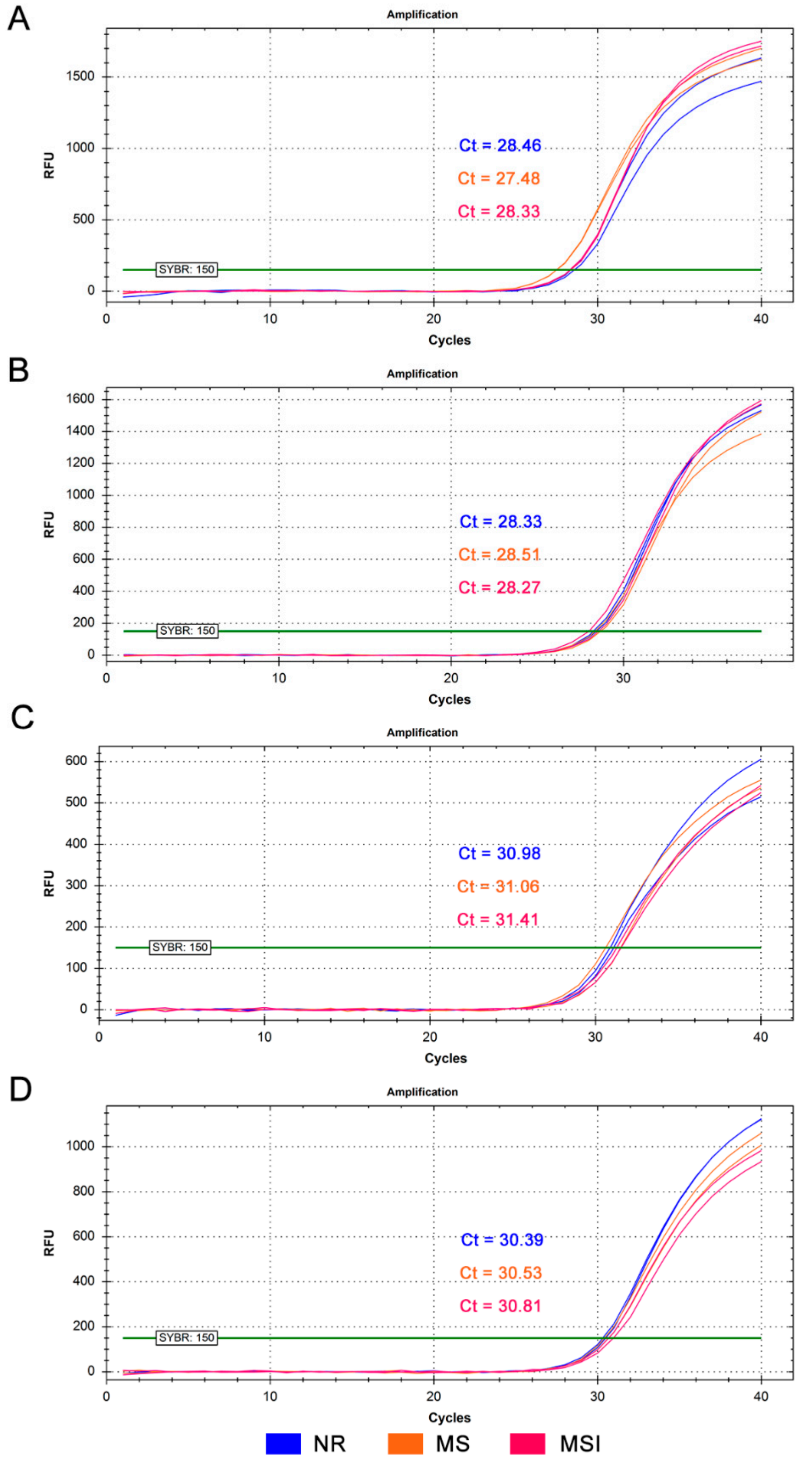

Supplementary Figure S15. PCR amplification curve of *sigA* under NR, MS, and MSI conditions from Mtb H37Rv (A), INH-R (B), RIF-R (C), and MDR (D). The result demonstrates that the expression of *sigA* was presented as an equal level under three conditions and can be used as a reference gene.
